# Using Advanced Bioinformatics Tools to Identify Novel Therapeutic Candidates for Proliferative Vitreoretinopathy

**DOI:** 10.1167/tvst.12.5.19

**Published:** 2023-05-16

**Authors:** Edward F. Xie, Bingqing Xie, Urooba Nadeem, Mark D'Souza, Gonnah Reem, Dinanath Sulakhe, Dimitra Skondra

**Affiliations:** 1 Chicago Medical School, Rosalind Franklin University of Medicine and Science, Chicago, IL, USA; 2 Department of Medicine, The University of Chicago, Chicago, IL, USA; 3 Department of Pathology, The University of Chicago, Chicago, IL, USA; 4 Center for Research Informatics, The University of Chicago, Chicago, IL, USA; 5 Department of Ophthalmology and Visual Science, The University of Chicago, Chicago, IL, USA

**Keywords:** proliferative vitreoretinopathy, PVR, bioinformatics, drug repurposing, drug therapy

## Abstract

**Purpose:**

Proliferative vitreoretinopathy (PVR) is the dreaded cause of failure following retinal detachment repair; however, no cures or preventative therapies exist to date. The purpose of this study was to use bioinformatics tools to identify drugs or compounds that interact with biomarkers and pathways involved in PVR pathogenesis that could be eligible for further testing for the prevention and treatment of PVR.

**Methods:**

We queried PubMed to compile a comprehensive list of genes described in PVR to date from human studies, animal models, and genomic studies found in the National Center for Biotechnology Information database. Gene enrichment analysis was performed using ToppGene on PVR-related genes against drug–gene interaction databases to construct a pharmacome and estimate the statistical significance of overrepresented compounds. Compounds with no clinical indications were filtered out from the resulting drug lists.

**Results:**

Our query identified 34 unique genes associated with PVR. Out of 77,146 candidate drugs or compounds in the drug databases, our analysis revealed multiple drugs and compounds that have significant interactions with genes involved in PVR, including antiproliferatives, corticosteroids, cardiovascular agents, antioxidants, statins, and micronutrients. Top compounds, including curcumin, statins, and cardiovascular agents such as carvedilol and enalapril, have well-established safety profiles and potentially could be readily repurposed for PVR. Other significant compounds such as prednisone and methotrexate have shown promising results in ongoing clinical trials for PVR.

**Conclusions:**

This bioinformatics approach of studying drug–gene interactions can identify drugs that may affect genes and pathways implicated in PVR. Predicted bioinformatics studies require further validation by preclinical or clinical studies; however, this unbiased approach could identify potential candidates among existing drugs and compounds that could be repurposed for PVR and guide future investigations.

**Translational Relevance:**

Novel repurposable drug therapies for PVR can be found using advanced bioinformatics models.

## Introduction

Proliferative vitreoretinopathy (PVR) is an aberrant wound healing response following retinal detachment (RD) repair that is characterized by the proliferation of contractile cellular membranes in the vitreous cavity and retinal surfaces. These proliferative membranes arise in response to an influx of cytokines, inflammatory growth factors, and inflammatory cells from the systemic circulation after breakdown of the blood–retinal barrier. PVR can affect 5% to 10% of patients with RD and remains the most common cause of failure and blindness following the surgical repair of RD.^1^ Although surgery remains the primary method for treating PVR, the primary success rate at 6 months after surgery falls around 60% to 75% despite advancements in surgical technique, and PVR is associated with cases with recurrent RDs and poor outcomes.^1^^–^^3^

Several pharmacologic interventions that target inflammation, cell proliferation, and fibrosis have been tested for the treatment and prevention of PVR, albeit without long-term success. Steroids, anti-vascular endothelial growth factor (VEGF) agents, and antineoplastic agents have shown positive results in preclinical models, but most of these therapies have failed to demonstrate efficacy in prospective human clinical trials. For example, a recent clinical trial for PVR prevention showed that corticosteroid use in the form of injections and implants failed to yield significant preventative effects or impact visual outcome.^4^^,^^5^ Daunorubicin, an antiproliferative compound, was also investigated for PVR prevention; although it did reduce vitreoretinal reoperation in patients with PVR after 1 year, it failed to significantly impact the retinal reattachment rate and visual acuity after 1 year.^6^

Despite the lack of a pharmacological agent with positive clinical trial results, the use of adjunctive therapies to prevent cellular proliferation holds promise for PVR prevention or recurrence after surgery.^7^ To date, the failed clinical studies required the administration of medicines via a perisurgical application or as a postsurgery intravitreal bolus injection. We reason that a nontoxic, well-tolerated, oral medication may better manage or prevent PVR. For a complex, multifactorial disease such as PVR, the traditional approach of identifying a singular target for a disease may be challenging and less likely to succeed. Systems medicine can study the interaction of multiple networks to singularize an output and formulate a novel solution. By using bioinformatic models to piece together computational predictions, high-throughput lab data, and prior knowledge from primary databases, we can take an unbiased approach toward discovering new therapeutic targets for PVR. This systems medicine strategy has previously discovered potential therapeutic targets for other multifactorial disorders such as refractory epilepsy, asthma, various cancers, and Alzheimer's disease, and it has recently been used by our group for retinal conditions such as age-related macular degeneration (AMD) and retinal neuroprotection.^8^^–^^15^

Unfortunately, the development of a single new drug for clinical applications is a costly and laborious process, but this is an issue that network medicine can address by identifying previously approved drugs and repurposing them for novel indications. Using a network-based approach to identify repurposable drugs offers a less costly and more efficient approach to recognizing candidates for new indications for drugs and compounds with appreciable safety profiles and pharmacokinetic/pharmacodynamics data. As this approach allows the focus to fall on existing U.S. Food and Drug Administration (FDA)-approved drugs with extensive clinical data and usage, testing and data collection become more readily accessible. Based on the use of a network-centric method to evaluate all of the described genes in PVR to date, we hypothesized that we could identify novel chemicals and known drugs for PVR indications.

## Methods

### Literature Search and Data Extraction

We queried studies from the NCBI database (https://www.ncbi.nlm.nih.gov/gene/) to aggregate a list of genes related to PVR (as of August 2022). After evaluating the abstracts from our initial query of published PubMed studies from PVR animal models, human studies, and genetic association studies, we included only the studies that showed a significant association between PVR and the proposed gene pathways and genomic biomarkers. The frequency of false-positive findings was reduced by excluding articles demonstrating weak or insignificant relationships. We examined the whole texts of select articles to ensure that their content corroborated the findings. Genes that were reported to be strongly associated with PVR in these studies were chosen for this investigation. Because neither humans nor animals were used in this investigation, ethical approval was not required.

### Discovering Potential PVR Therapeutic Targets Via Enrichment Analysis

The degree of association between the candidate PVR target genes and molecules and associated drugs was analyzed to identify potential PVR drugs on the basis that a drug with a greater association to its corresponding disease genes will work more effectively.^16^ For this reason, drugs identified from an enrichment analysis of chemical–gene association databases that interact specifically and strongly with the PVR genes can potentially be used as therapeutic targets.

We entered the gene dataset obtained from our PubMed query into the ToppFun function of the ToppGene Suite (http://toppgene.cchmc.org/) to carry out a gene set enrichment analysis. To create a pharmacome for predicting drug–gene interactions, ToppGene gathered 22,832 genes and a total of 77,146 drug annotations from five different sources: Broad Institute CMap Up, Broad Institute CMap Down, Comparative Toxicogenomics Database (CTD), Drug Bank, and Stitch. To find the drug with the strongest connections, ToppGene created a representative profile from the inputted genes and compared it to its 22,832 test set genes. In order to evaluate statistical significance, a hypergeometric distribution with Bonferroni adjustment was used. In this analysis, medications with a false discovery rate (FDR)-adjusted *P* value of 0.05 were taken into consideration.

### Selection of Drugs and Chemicals Useful in PVR

Compounds with unclear clinical indications or known damaging effects to human health such as particulate matter, ozone, and asbestos were carefully filtered out from the final list. As the pharmacome was generated using multiple databases, there are also redundant compounds found in the final analysis that are essentially combined by keeping the compound with the highest *P* value.

### Visualization of the Networks

Using Cytoscape, we visualized the relationships between the curated list of PVR genes and enriched pharmaceutical compounds. The drug–gene network follows a prefuse force directed layout based on the edge betweenness centrality measure.^17^ Nodes representing drugs in this graphical layout were shown in red, and nodes representing genes were shown in green. To represent prospective hub nodes, which are nodes with comparably more connections to nearby nodes, nodal size was correlated with the closeness centrality hub, and so the largest nodes corresponded to the highest closeness. The visualization did not include genes with fewer than three drug associations.

A gene–pathway network was also created from the functional enrichment of the PVR genes. Enrichment analysis for Gene Ontology (GO), disease, and pathway databases was performed with the Lynx database system.^18^ For the creation of a gene–pathway network, 10 of the most significant pathways from the enrichment analysis were chosen along with their associated PVR genes. We again used Cytoscape to visualize and analyze the network with a prefuse force directed layout based on edge betweenness, with nodal size representing closeness centrality.^17^ Drug nodes were once again colored green, but the pathway nodes were colored blue.

## Results

We identified 34 unique genomic and proteomic biomarkers associated with PVR from our PubMed gene query. Enrichment analysis of the curated PVR gene list generated 7884 chemical compounds from the drug–gene database with an FDR adjusted *P* cutoff of 0.05. The initial list of compounds included redundant drugs, as our pharmacome was generated using multiple drug databases from ToppGene, which includes toxic compounds (i.e., asbestos, H_2_O_2_), as well. After manually filtering out redundant and deleterious drugs, we compiled a snapshot of the top 50 most significant compounds associated with PVR biomarkers with potential for drug repurposing (Table 1).

**Table 1.** Top 50 Filtered Drugs Targeting PVR Genes Predicted by ToppGene Database in Order of *P* Value

Filtered Position	Unfiltered Position	Compound	Source	*P*	*q*-Value FDR (Benjamini–Hochberg)	Hit Count in Query List
1	8	U 0126	CTD	5.12E-19	1.11E-15	16
2	10	Heparin	CTD	8.53E-19	1.48E-15	12
3	11	Simvastatin	CTD	9.76E-19	1.54E-15	17
4	12	Cobalt	CTD	1.21E-18	1.75E-15	14
5	16	SB 203580	CTD	3.01E-18	3.27E-15	15
6	20	Aspirin	CTD	1.30E-17	1.13E-14	17
7	24	Curcumin	CTD	2.03E-17	1.47E-14	18
8	26	Enalapril	CTD	3.70E-17	2.47E-14	11
9	27	Luteolin	CTD	4.03E-17	2.59E-14	11
10	29	Carvedilol	CTD	5.65E-17	3.38E-14	11
11	31	Capsaicin	CTD	9.17E-17	5.14E-14	15
12	35	Gallic acid	CTD	1.43E-16	7.11E-14	10
13	37	Amifostine	CTD	1.62E-16	7.58E-14	7
14	41	Actinomycin D	Stitch	2.71E-16	1.15E-13	15
15	42	Furosemide	CTD	4.64E-16	1.87E-13	11
16	44	Galangin	CTD	5.26E-16	2.05E-13	9
17	47	Taxol	Stitch	7.02E-16	2.60E-13	15
18	49	Emodin	CTD	8.43E-16	2.94E-13	11
19	53	Diclofenac	CTD	9.11E-16	2.95E-13	15
20	57	Octreotide	CTD	1.13E-15	3.39E-13	8
21	59	Myricetin	CTD	1.33E-15	3.92E-13	9
22	61	Propionyl carnitine	CTD	1.43E-15	4.07E-13	6
23	62	Deferoxamine	CTD	1.89E-15	5.30E-13	11
24	67	Genistein	Stitch	2.36E-15	6.12E-13	18
25	72	Celecoxib	CTD	3.62E-15	8.73E-13	13
26	73	Ascorbic acid	CTD	3.70E-15	8.80E-13	15
27	77	Thymoquinone	CTD	5.11E-15	1.15E-12	8
28	88	Kaempferol	CTD	1.18E-14	2.34E-12	10
29	89	Telmisartan	CTD	1.48E-14	2.89E-12	9
30	90	*N*-acetylcysteine	Stitch	1.52E-14	2.94E-12	14
31	91	Hesperetin	CTD	1.56E-14	2.98E-12	8
32	92	Bleomycin	CTD	1.70E-14	3.20E-12	13
33	98	Fisetin	CTD	2.41E-14	4.27E-12	8
34	99	Doxorubicin	CTD	2.62E-14	4.60E-12	20
35	103	Thioctic acid	CTD	2.94E-14	4.97E-12	10
36	104	Losartan	CTD	3.33E-14	5.52E-12	10
37	110	Curdlan	CTD	4.37E-14	6.90E-12	9
38	112	Naringenin	CTD	5.17E-14	8.01E-12	9
39	114	Salvianolic acid B	CTD	5.48E-14	8.35E-12	7
40	115	Betamethasone	Stitch	5.62E-14	8.49E-12	18
41	117	Pravastatin	CTD	6.10E-14	9.05E-12	8
42	119	Baicalin	CTD	6.80E-14	9.92E-12	7
43	120	Chrysin	CTD	7.16E-14	1.04E-11	9
44	135	Ursodeoxycholic acid	CTD	1.48E-13	1.90E-11	11
45	143	Docetaxel	CTD	2.72E-13	3.26E-11	9
46	144	Ellagic acid	CTD	2.72E-13	3.26E-11	9
47	148	Carbocysteine	CTD	3.01E-13	3.53E-11	5
48	151	Apigenin	CTD	3.27E-13	3.77E-11	10
49	157	Indomethacin	Stitch	3.70E-13	4.10E-11	14
50	158	Benazepril	CTD	4.01E-13	4.38E-11	8

**Figure 1.**
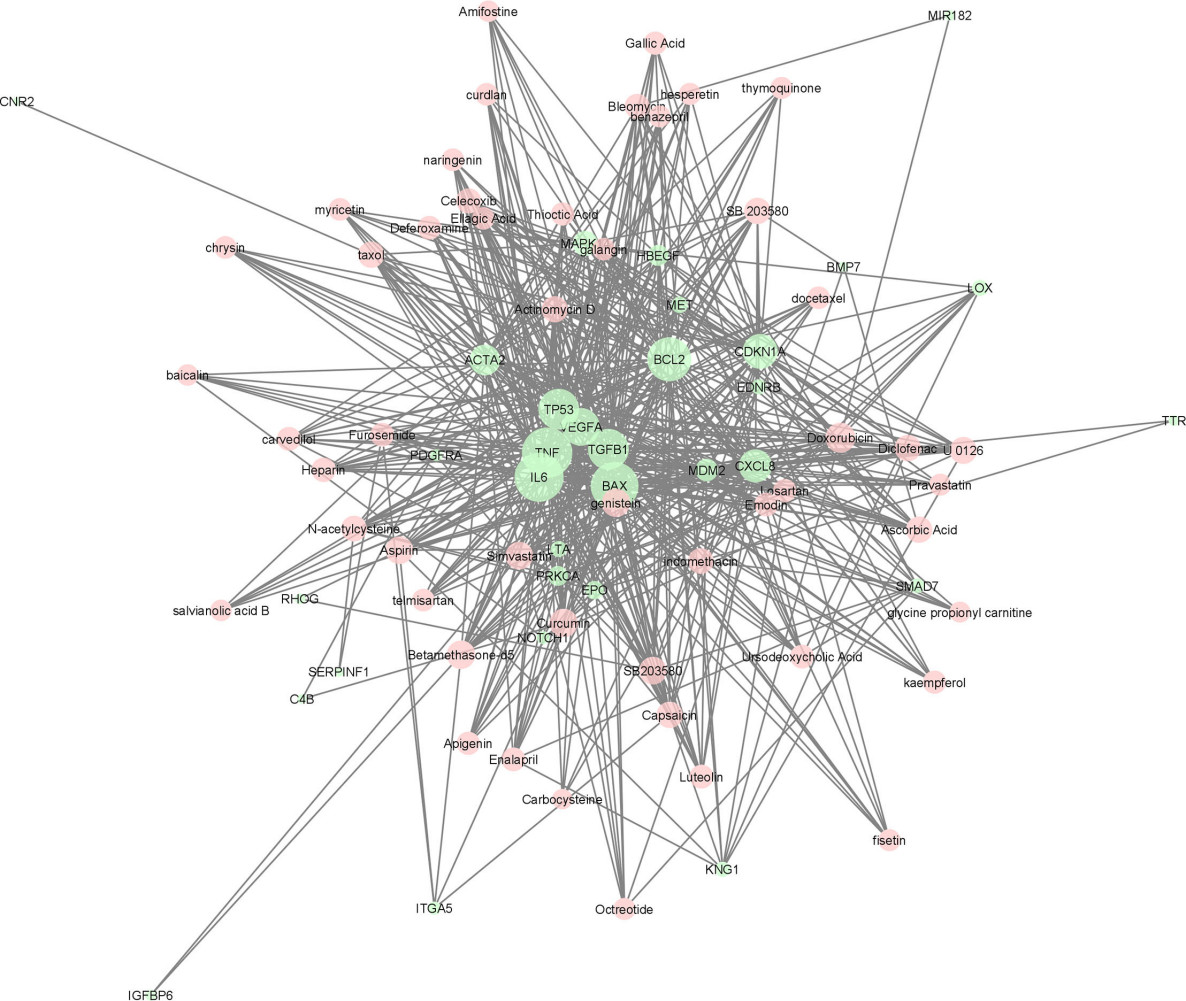
Force-directed graph of drug–gene interactions. Node size and edges are represented based on centrality metrics analysis. Hub nodes that have the greatest number of associations with other nodes are larger and found in the center. Drugs are shown in *red*, genes in *green*.

Many of the statistically significant compounds enriched in our analysis affect key biologic pathways implicated in PVR pathogenesis including inflammation, aberrant cellular proliferation, and cellular migration. The drug classes identified from the PVR pharmacome include antioxidants (e.g., *N*-acetylcysteine, ascorbic acid, glutathione, alpha-lipoic acid, coenzyme Q10), polyphenol micronutrients (e.g., curcumin, apigenin, pterostilbene, naringin), lipid-lowering agents (e.g., simvastatin, atorvastatin), and cardiovascular agents (e.g., nifedipine, losartan, carvedilol, enalapril). Among the top compounds, doxorubicin, curcumin, genistein, simvastatin, and betamethasone have the greatest number of associations with the list of PVR genes as reflected by their closeness centrality values (Fig. 1). Polyphenol micronutrients, such as curcumin (*P* = 1.66 × 10^−51^) and apigenin (*P* = 3.27 × 10^−13^), are a class of compounds enriched in our analysis that exhibit combined antioxidative and anti-inflammatory activity. Curcumin, the primary biologically active ingredient in turmeric, is the most significant polyphenol ingredient, as it was found to affect 18 of the 32 genes. Apigenin is associated with 10 of the 34 PVR genes.

Several significant compounds in the analysis such as corticosteroids and antimetabolites exhibit direct antiproliferative and anti-inflammatory activity, and some are currently in use clinically. Corticosteroids, including betamethasone (*P* = 5.62 × 10^−14^) and prednisone (*P* = 8.92 × 10^−^^7^), are significantly associated with PVR genes and affect 18 and four of the total 34 PVR genes, respectively. Additionally, we identified many antimetabolites among the most significant compounds, including 5-FU (*P* = 6.14 × 10^−13^), daunorubicin (*P* = 5.01 × 10^−13^), doxorubicin (*P* = 2.62 × 10^−14^), dactinomycin (*P* = 2.71 × 10^−16^), and methotrexate (*P* = 1.70 × 10^−4^), which affect 13, 10, 20, 15, and five of the PVR genes, respectively.

We also identified many commonly prescribed drugs with well-established safety profiles that are approved for various indications. These medications include simvastatin (*P* = 9.76 × 10^−19^), which affects 17 of the 34 PVR genes and belongs to the most commonly prescribed drug class for hyperlipidemia, and cardiovascular agents, such as the ACE inhibitor enalapril (*P* = 3.70 × 10^−17^) and the beta blocker carvedilol (*P* = 5.65 × 10^−17^), both of which affect 11 of the 34 PVR genes and are two of the most prescribed drug classes for heart failure and hypertension. Metformin (*P* = 8.30 × 10^−12^), which affects 11 of the 34 PVR genes, is the most used drug for type 2 diabetics and was found in a list of the top 100 filtered compounds (Supplementary Table S1). Investigational compounds, including the MEK inhibitor U0126 (*P* = 5.12 × 10^−19^) and the MAP kinase inhibitor SB203580 (*P* = 3.01 × 10^−18^), are also significantly associated with PVR genes; however, these compounds are poorly understood and are experimental in nature.

**Figure 2.**
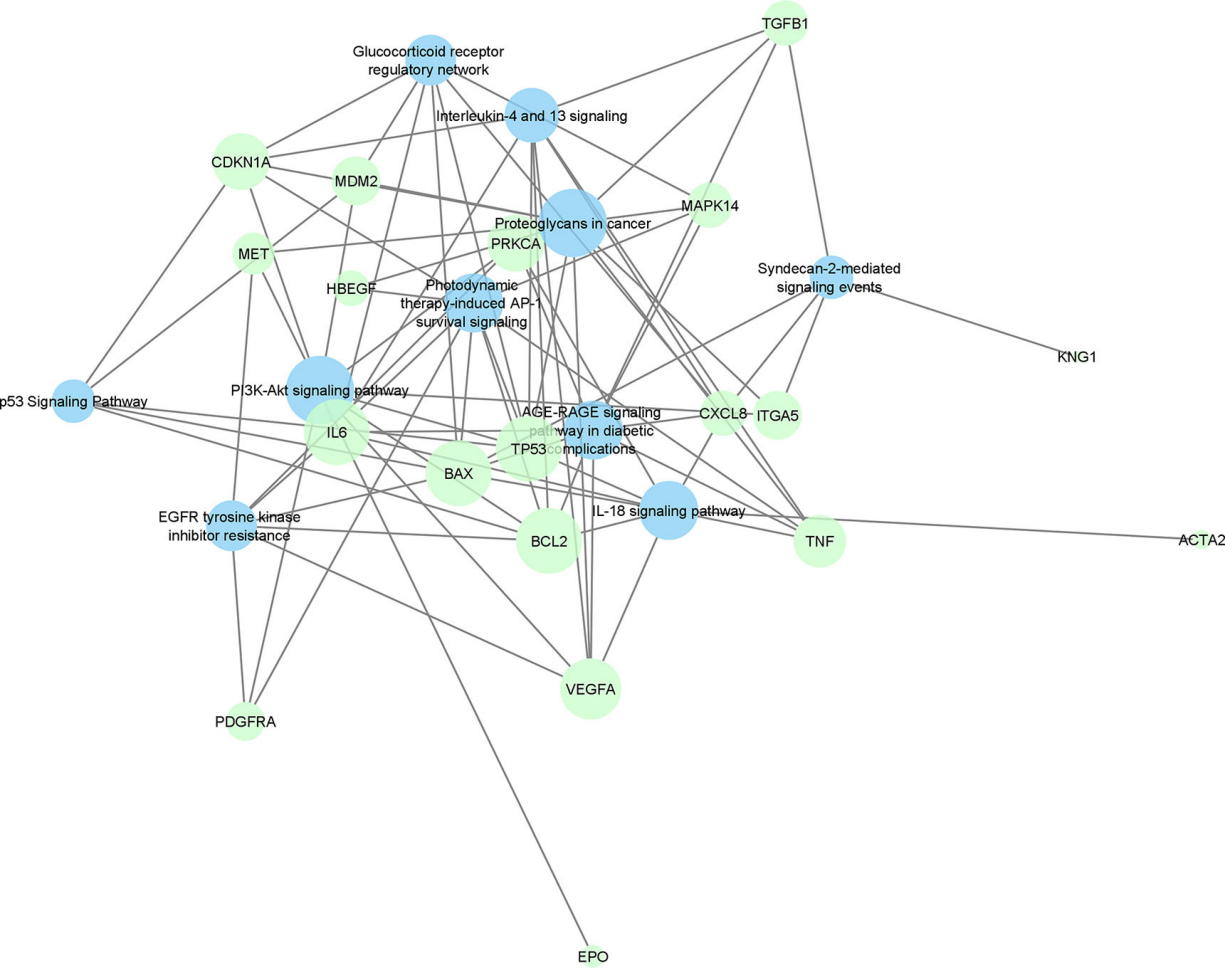
Force-directed graph of drug–gene interactions. Hub nodes that have the greatest number of associations with other nodes are larger and found in the center. Node size and edges are represented based on centrality metrics analysis. Pathways are shown in *blue*, genes in *green.*

Analysis of the PVR gene list with the Lynx Enrichment tool revealed top gene pathways such as “photodynamic therapy-induced AP-1 survival signaling,” “proteoglycans in cancer,” “AGE-RAGE signaling pathway in diabetic complication,” “interleukin-4 and 13 signaling,” “p53 signaling pathway,” “PI3K/Akt signaling pathway,” “EGFR tyrosine kinase inhibitor resistance,” “glucocorticoid receptor regulatory network,” “syndecan-2 mediated signaling events,” and “interleukin-18 signaling pathway” (Fig. 2). In this drug–pathway network, the apoptotic regulator genes including Bcl-2-like protein 4 (*BAX*), B-cell lymphoma 2 (*Bcl-2*), and *TP53*, along with the pro-inflammatory cytokine interleukin-6 (*IL-6*), are the genes that hold the highest degree value. The top pathway by significance, “photodynamic therapy-induced AP-1 survival” (*P* = 3.38 × 10^−15^), is associated with these four top genes as well as five additional genes, including *CDKN1A*, *MAPK14*, *PDGFRA*, *TNF*, and *HBEGF*. These nine genes make up the primary targets for the top drugs identified in our PVR pharmacome (Fig. 1). Gene ontology term analysis indicates that these nine genes are most associated with “blood vessel development” and “regulation of cell migration” (Table 2). Supplementary Table S2 provides the complete list of genes, drugs, gene–drug connections, and number of genome hits.

**Table 2.** Most Significant Gene Ontology Description of Genes Associated With Genes Related to PVR

ID	Function	*P*	*q*-Value FDR (Benjamini–Hochberg)	Hit Count in Query List	*q-*Value FDR (Benjamini–Yekutieli)
GO:0001568	Blood vessel development	1.24E-18	4.18E-15	21	3.70E-14
GO:0030335	Positive regulation of cell migration	2.44E-18	4.18E-15	18	3.70E-14
GO:2000147	Positive regulation of cell motility	5.10E-18	4.18E-15	18	3.70E-14
GO:0001944	Vasculature development	5.38E-18	4.18E-15	21	3.70E-14
GO:0051272	Positive regulation of cellular component movement	8.31E-18	4.73E-15	18	4.18E-14
GO:0040017	positive regulation of locomotion	8.51E-18	4.73E-15	18	4.18E-14
GO:0033993	Response to lipid	2.23E-17	9.63E-15	22	8.52E-14
GO:0035295	Tube development	5.71E-17	1.97E-14	23	1.75E-13
GO:0001932	Regulation of protein phosphorylation	1.98E-16	5.91E-14	21	5.23E-13
GO:0051094	Positive regulation of developmental process	2.34E-16	6.49E-14	22	5.74E-13
GO:0072359	Circulatory system development	3.08E-15	7.09E-13	22	6.27E-12
GO:1902533	Positive regulation of intracellular signal transduction	3.19E-15	7.09E-13	19	6.27E-12
GO:0009611	Response to wounding	1.03E-14	1.81E-12	17	1.60E-11
GO:0014070	Response to organic cyclic compound	2.32E-14	3.61E-12	20	3.19E-11
GO:0048514	Blood vessel morphogenesis	4.73E-14	6.18E-12	17	5.47E-11
GO:0043067	Regulation of programmed cell death	6.93E-14	8.16E-12	21	7.22E-11

## Discussion

Proliferative vitreoretinopathy is the primary cause of failure leading to poor visual outcomes after retinal detachment surgery. Despite advancements in RD surgery and increased understanding of the pathophysiology underlying PVR, there is no definitive solution to the problem of treating this multifactorial process. Any disease process reflects a disruption of multiple elements in a previously well-balanced biologic system. A network medicine approach can help definitively reveal the molecular connections across grossly distinct pathways to construct biological relationships. Until recently, the identification of patients at high risk for PVR has been dependent on the analysis of clinical data and inflammatory markers that yield predictive formulas with poor sensitivity and specificity values.^19^^,^^20^ The Retina 4 project, a case-controlled gene association study, launched a novel approach of using machine learning techniques to study the genetic components of PVR and to develop more accurate predictive models.^21^ With the recent advances in multiomics, it has become possible to understand large-scale biological networks that can be exploited to help us unravel complex processes such as PVR. To predict the best therapeutic options, we employed a network medicine approach in this study to identify links among known PVR genes, molecules, and pathways and available pharmacological drugs.

Our study identified several antiproliferative agents associated with PVR agents such as 5-FU, daunorubicin, doxorubicin, taxol, colchicine, and methotrexate. Although antiproliferative agents such as daunorubicin and 5-FU have shown promise in PVR animal models, they have produced inconsistent results in clinical studies. Daunorubicin is safe to use perioperatively and can reduce reoperation rates for patients with PVR undergoing retinal surgery, but patients who received this treatment experienced no difference in visual acuity and reattachment rate after 1 year.^6^ Similarly, 5-FU therapy in PVR detachment did not generate any significant improvements in anatomical or visual outcomes and produced worse visual outcomes for certain patients with macular-sparing PVR retinal detachments.^22^ Taxol and colchicine are two drugs that stabilize and inhibit microtubule formation, which reduces cellular migration and proliferation. Both compounds have demonstrated efficacy at preventing in vitro and in vivo models of PVR by inhibiting migration and proliferation of retinal pigment epithelial (RPE) cells, astrocytes, and fibroblasts; however, clinical studies have yet to be completed.^23^

Currently, methotrexate (MTX), another antineoplastic agent commonly used for chronic inflammatory disorders such as rheumatoid arthritis and psoriasis, remains one of the few promising pharmaceutical compounds that is currently being used clinically for PVR with ongoing clinical trials. Initial in vitro models have shown that methotrexate can protect against PVR by inhibiting cell proliferation and regulating apoptosis.^24^ Intravitreal injections of MTX have demonstrated safety in silicone-filled eyes and shown efficacy in improving the postoperative visual acuity of PVR eyes in one pilot study.^25^ One recent retrospective study showed that intravitreal methotrexate infusion at the time of pars plana vitrectomy for retinal detachment repair was associated with a lower incidence of PVR development in high-risk patients.^26^ Repeated intravitreal injections of MTX in the postoperative period correlate with improved anatomical outcomes, and serial injections as an adjunct to surgical repair continue to be tested in postoperative PVR eyes.^27^ Our analysis revealed that “photodynamic therapy-induced AP-1 survival” was the most significant pathway associated to our PVR gene list from our analysis, and MTX exposure in mice has been shown to increase activator protein-1 (AP-1)-dependent transcription.^28^ This correlation points to one possible mechanism in which MTX can suppress inflammation and cellular proliferation in PVR. As the GO terms of the genes involved in this AP-1 pathway are associated with “blood vessel development” and “regulation of cell migration,” it is possible that the development of proliferative, contractile membranes in PVR may be similarly driven by an AP-1–mediated tumor cell response pathway that is targeted by MTX.

Corticosteroids, the earliest tested pharmaceutical compounds for PVR, were also identified in our study. Intravitreal steroids such as triamcinolone acetonide (TA) and dexamethasone have shown safety and efficacy in many randomized clinical trials studying posterior segment diseases^29^^,^^30^; however, they have yielded inconsistent results in PVR clinical trials.^31^ Although many hospitals continue to use dexamethasone or TA during RRD surgery to reduce the risk of PVR, studies on intravitreal dexamethasone implants and triamcinolone injections as adjunctive therapy failed to show significant improvements in visual outcomes for patients undergoing vitrectomy.^5^^,^^32^ However, one prospective randomized placebo-controlled clinical trial of systemic corticosteroids did show that prednisone started prior to RD surgery can reduce the incidence of early stages of PVR such as cellophane membrane formation after surgery.^33^ A meta-analysis by Shi et al.^34^ studying corticosteroids as an adjunct to RRD surgery also found a decrease in postoperative PVR incidence rate especially for grade B PVR. Unfortunately, extraneous factors such as drug delivery, surgeon skill, steroid dosages, and surgical methods all impact the results of these studies. Efforts to create a sustained intraocular delivery system with combination drug therapy using both a corticosteroid and antineoplastic have yielded promising results in an vivo rabbit model and have also shown that dual therapy works better than single therapy with a corticosteroid or antineoplastic agent.^35^

We also identified various antioxidative micronutrients in the list of the top over-represented compounds associated with PVR genes. One of the primary initiating factors in PVR is the disruption of the blood–retinal barrier leading to an influx of growth factors and chemokines into the vitreous cavity. Key agents in the pathogenesis of PVR that concurrently serve as PVR biomarkers include platelet-derived growth factor (PDGF), VEGF, epidermal growth factor (EGF), transforming growth factor beta (TGF-β), tumor necrosis factor alpha (TNF-α), interferon beta, and IL-8. Three of the top antioxidative micronutrients that we have identified (i.e., curcumin, apigenin, and *N*-acetylcysteine) are all known inhibitors of the nuclear factor kappa B (NF-κB) transcription factor and can modulate levels of proangiogenic growth factors such as IL-8, VEGF, and TNF-α in the eye.^36^^–^^38^ Curcumin and apigenin have also been shown to inhibit RPE cellular proliferation in vitro by inducing caspase 3-/7-dependent cell death and reducing EGF expression.^39^^–^^41^ *N*-Acetylcysteine also suppress cell proliferation in vitro and blocks retinal detachment in rabbits via suppression of PDGF-R.^42^ Of note, apigenin has exhibited a protective effect against oxidative stress and liver and kidney damage induced by methotrexate in mice, which opens up the interesting possibility of polypharmacy for the clinical treatment of PVR using methotrexate today.^43^ Currently, bioavailability stands as a significant impediment for all nutritional supplement drugs, but alternative drug delivery methods such as curcumin-loaded scleral plugs have demonstrated efficacy in decreasing proinflammatory cytokines in the eye.^44^ Novel formulations such as oral Longvida Optimized Curcumin have been shown to accumulate in the retina of human subjects and are being trialed in patients with dry AMD for drusen regression and to reduce choriocapillaris density loss.^45^ Ultimately, further studies on drug delivery and pharmacokinetics are still necessary to perfect the usage of antioxidants and nutrients for PVR.

Our study also identified some newly identified candidates that are FDA-approved agents with significant associations to risk genes of PVR, such as metformin, which could be repurposed for PVR. Metformin is the first-line agent for type 2 diabetes and has additional off-label uses for various cancers and cardiovascular disease due to its antioxidative, antiproliferative, and anti-inflammatory effects.^46^ The pathogenesis of both PVR and proliferative diabetic retinopathy (PDR) is similarly defined by the breakdown of the blood–retina barrier with leakage of proinflammatory cytokines into the vitreous, so it is hypothesized that anti-diabetic drugs such as metformin may have preventive and therapeutic roles in PVR. There is also evidence that metformin can suppress retinal angiogenesis and inflammation, which are pathogenic features in both diabetic retinopathy and PVR.^47^ In the human vitreous of both controls and patients with PDR, patients with long-term metformin treatment had lower concentrations of various inflammatory cytokines and chemokines such as intercellular adhesion molecule 1 (ICAM-1), TNF-α, IL-8, and monocyte chemoattractant protein-1 (MCP-1).^48^ In mouse models of retinal detachment, subocular injections of insulin and insulin sensitizers such as metformin can attenuate the post-detachment inflammatory response and leukocyte infiltration associated with cone loss.^49^ Although metformin has yet to be trialed as a therapeutic option for PVR in humans, this anti-diabetic medication has certainly proven its antiproliferative and anti-inflammatory effect in the eye and may deserve a closer look in the future.

Another class of FDA-approved agents with promising results for inhibiting the development of PVR are statins, 3-hydroxy-3-methylglutaryl coenzyme A (HMG-CoA) reductase inhibitors that are used as lipid-lowering agents. In PVR, the formation and contraction of proliferative membranes cause retinal breaks and redetachment of the retina. Statins inhibit the mevalonate pathway, which reduces cholesterol synthesis, and the Rho/Rho-kinase pathway, which is critical for smooth muscle contraction and stress fiber formation in non-muscle cells.^50^^–^^52^ Intravitreal injections of simvastatin in rabbits reduced the cicatricial contraction of proliferative membranes and lowered concentrations of TGF-β2, which is a fibrogenic cytokine overexpressed in patients with PVR that activates Rho-kinase.^53^ In a Finnish population-based cohort study, the use of simvastatin and atorvastatin was associated with a significantly lower risk of re-vitrectomy in eyes that were operated on for RD.^54^ Our data further support evidence that statin use may have the potential to affect the outcomes of re-vitrectomy rates after RD. One recent pharmacokinetics study on the intravitreal delivery of statins also found that the therapeutic statin concentration that alleviates inflammation induced by bacterial lipopolysaccharide in vitro can be maintained with existing drug delivery technologies.^55^ Nevertheless, both retrospective large-scale studies on the incidence of PVR in patients taking statins and larger clinical trials are necessary to discern the precise effect of these drugs on the pathology.

Commonly used cardiovascular agents, including the angiotensin-converting enzyme (ACE) inhibitor enalapril and the beta blocker carvedilol, are also top candidates found in our analysis. The induction of fibrosis by the renin–angiotensin system is evident in multiple organ systems, including the eye, and the release of profibrotic cytokines and growth factors is common in the pathogenesis of both PVR and renal fibrosis.^56^ Similar to how TGF-β induces the epithelial-to-mesenchymal transition (EMT) of RPE cells into contractile myofibroblasts in PVR, TGF-β induces EMT of renal tubular epithelial cells in renal fibrosis.^57^^,^^58^ However, despite inhibition of TBF-β expression and fibrosis in the kidney with systemic ACE inhibitors, a similar dosage with ACE inhibitors in a primary human study did not significantly decrease the levels of cytokines and growth factors such as TGF-β, VEGF, IL-6, or PDGF in the aqueous humor of patients with RD or affect the incidence of PVR development.^59^ Nonetheless, it is unclear if the systemic intake and accumulation of ACE inhibitors in the eye have a direct effect, and the pursuit of a more direct ocular delivery route remains an attractive option for future experiments. Similar to ACE inhibitors, beta blockers (especially carvedilol and metoprolol) also suppress renin secretion and angiotensin II formation.^60^ Additionally, carvedilol is widely used clinically for chronic heart failure and following myocardial infarction in part due to its significant suppression of myocardial fibrosis and cardiac remodeling, and this effect is further enhanced when carvedilol is used in combination with enalapril, as shown in the CARMEN trial.^61^ Although the antifibrotic activity of carvedilol is not well understand, it has shown antifibrotic activity in the liver of rats by overexpressing both microRNA-200a, which modulates EMT markers, and SMAD7, which attenuates TBF-β and other inflammatory markers.^62^ Both classes of cardiovascular agents require additional preclinical and clinical trials to further assess their antifibrotic effects in the eye and to fine-tune the dosing and delivery.

In this study, we focused on drugs with antiproliferative and antioxidative agency based on hub genes identified from our enrichment analysis that play a role in regulation of cell proliferation and migration per their GO annotations. BAX, Bcl, and TP53 are apoptotic regulators that play a role in tumor progression by carefully balancing the pro-apoptotic protein BAX and the anti-apoptotic protein Bcl-2. IL-6 is a proinflammatory cytokine that is elevated in PVR eyes and causes a dose-dependent elevation of RPE cell proliferation.^63^ The top pathway by significance, “photodynamic therapy-induced AP-1 survival,” refers to a tumor cell stress response mediated by the AP-1 transcription factor that promotes cellular survival after stimulation by ROS-mediated oxidative stress and damage. This pathway is also connected to five additional genes, including *CDKN1A*, *MAPK14*, *PDGFRA*, and *HBEGF*, in addition to the four top genes, *BAX*, *BCL*, *IL-6*, and *TP53*, which were previously mentioned. Review of the gene ontology term analysis indicates that these nine genes are most associated with “blood vessel development” and “regulation of cell migration,” suggesting that development of proliferative, contractile membranes in PVR may be similarly driven by this AP-1–mediated tumor cell response pathway. As oxidative stress is a well-known inducer of cellular proliferation through stimulation of mitogenic pathways, recent studies on neurodegenerative and vasoproliferative disorders are focusing on the genetic factors responsible for how redox imbalance and may induce aberrant cellular proliferation.^64^^,^^65^ Future studies to characterize the genetic basis of PVR may enhance prospective inquiries into robust pharmacological candidates. Additional insight into how genetic polymorphisms influence susceptibility to PVR can also improve targeted therapies.^66^^,^^67^

### Limitations

Unfortunately, there are impediments to creating an accurate model for a heterogeneous disease process such as PVR. Many pharmaceutical agents with positive results in preclinical models fail to show efficacy in large prospective human clinical trials. Even today, our understanding of the pathophysiology of PVR is likely deficient, and current animal models may not be adequately representative.^68^ Our incomplete understanding is reflected in the lack of a universal grading scheme for PVR, and more than 25% of PVR studies fail to include or reference a grading scheme.^69^ There are also inherent limitations for any in silico approach to drug discovery. The approach used in this study has several disadvantages. First, databases such as STITCH and CTD contain intrinsic errors, such as false positives and postulated chemical–gene linkages resulting from molecular activity downstream from the original chemical interactions; hence, determining the relative importance of each chemical–gene association is difficult. Second, genomics-based predictions rely on previously published research to discover drug–gene interactions, so the quantity of material available for each particular illness varies. For a rare, ocular pathology such as PVR, less research has been directed toward identifying biomarkers compared to more widespread diseases such as cancer or diabetes. Similarly, newer compounds or drugs with fewer clinical indications have a paucity of known drug–gene interactions and so may appear underrepresented in enrichment analyses compared to widely used FDA-approved drugs. Finally, we did not discern the impact on disease progression of the genes being either protective gene variants or risk gene variants. This study approach ultimately resembles a mea-analysis of hundreds of previous studies, and it is necessary to account for differences in data standards and experimental methodologies among the investigations. As there are data from animal models and transformed cell lines, certain projected drug–gene interactions unfortunately may not apply physiologically to humans.

## Conclusions

Our investigation revealed numerous medications and compounds that are relevant to PVR. From our analysis, we identified FDA-approved drugs such as enalapril, carvedilol, statins, metformin, and methotextrate, as well as micronutrients such as curcumin, apigenin, and *N*-acetylcysteine, that all have well-established pharmacodynamics and pharmacokinetics data. We believe that the proven safety and tolerability profiles of these drugs make them excellent candidates for repurposing. Furthermore, multifactorial pathologies such as PVR may require polypharmacy to synergistically tackle different disease pathways. A combination of different drug classes such as ACE inhibitors and beta blockers can have additive effects in cardiac fibrosis, and the same could be true in PVR. Meanwhile antioxidants such as apigenin promote anti-inflammatory and antiproliferative effects but also can directly reduce methotrexate toxicity. Just as a bioinformatics model utilizes open-source algorithms and databases to develop new tools for investigation, our systems biology methodology provides an impartial way to forecast novel therapeutic targets and permits an unbiased look at drug discovery. Conducting further studies using multiomics data regarding PVR will only increase the accuracy of predictive mathematical computer models of relationships between drugs and their targets. Ultimately, this study sheds light on the significance of a computational and bioinformatics approach in advancing our comprehension of complicated, multifactorial disorders such as PVR, and we hope our approach can be applied to drug discovery for other complex disorders in the future.

## Supplementary Material

Supplement 1

Supplement 2
